# Age-Friendly Design and Optimization of Digital Health Technologies for Medication Adherence in Older Adults With Chronic Diseases: Scoping Review

**DOI:** 10.2196/97466

**Published:** 2026-09-30

**Authors:** Liyuan Long, Lingjuan Zhou, Wenling Li, Penglin Chen, Jiulin Guo, Zhonglan Chen

**Affiliations:** 1 Department of Cardiology West China Hospital, Sichuan University Chengdu, Sichuan China; 2 West China School of Nursing Sichuan University Chengdu, Sichuan China; 3 Department of Clinical Pharmacy West China Hospital, Sichuan University Chengdu, Sichuan China; 4 Department of Information and Data Management West China Hospital, Sichuan University Chengdu, Sichuan China

**Keywords:** age-friendly, digital health technology, digital optimization, medication adherence, scoping review

## Abstract

**Background:**

Medication nonadherence represents a persistent clinical challenge among older adults with chronic diseases, complicating long-term disease management and prognosis. Digital health technologies (DHTs) deliver promising opportunities for remote, out-of-hospital medication management; nevertheless, age-related cognitive-physiological decline and insufficient digital literacy widen the digital divide, creating barriers to real-world uptake and sustained use of DHTs. Existing systematic reviews mainly synthesize intervention effect sizes, but few unpack multilevel drivers of mixed outcomes or generate actionable age-friendly design guidance that is empirically grounded and tailored to older adult needs.

**Objective:**

This scoping review aimed to identify and characterize DHT interventions designed to improve medication adherence in older adults with chronic diseases, examine the range of factors influencing their use, and derive age-friendly design principles from the available literature.

**Methods:**

Following the scoping review framework by Arksey and O’Malley and the PRISMA-ScR reporting standards, we searched 7 databases from January 1, 2013, to August 2026. An updated search was performed on September 2, 2026, to ensure the currency of the studies. Eligible sources included quantitative, qualitative, and mixed methods original studies as well as secondary reviews published in English or Chinese. Two independent reviewers performed screening, data extraction, and thematic synthesis guided by social cognitive theory and person-environment fit theory.

**Results:**

A total of 60 studies were included, of which 46 (76.7%) were published between 2022 and August 2026, indicating rapidly growing research interest. Study mapping revealed a skewed distribution across the medication journey, with most investigations concentrated on the daily routine phase, while prescription, pickup, storage, and refill remained severely understudied. We synthesized a 4-level model comprising individual, technical, social-environmental, and intervention program factors influencing DHT performance. Major study gaps were identified: only 1 study specifically recruited older adults living alone, only 1 study used validated standardized usability metrics, and no included study assessed bidirectional electronic health record interoperability. Based on barriers reported by older users, we derived a 5-dimension age-friendly design framework encompassing perceptibility, comprehensibility, operability, emotional warmth, and security-interoperability.

**Conclusions:**

Moving beyond prior reviews focused predominantly on intervention efficacy, this scoping review presents a theory-informed 4-level influencing-factor model and an empirically derived age-friendly design framework to guide future development and implementation. For clinical practice, nurses and other health care professionals should conduct preimplementation baseline assessments of older adults’ cognitive function, digital literacy, and social support and tailor DHT recommendations accordingly. For future research, investigators should prioritize socially vulnerable subgroups, including those living alone and those in resource-limited settings, standardize usability reporting using validated instruments, and actively integrate clinical system interoperability to enable closed-loop care. Closing intra–age-group digital health inequities requires interdisciplinary collaboration across nursing, medicine, pharmacy, and health informatics, ensuring that DHTs become tools of empowerment rather than sources of exclusion.

**Trial Registration:**

OSF Registries osf.io/za6f2; https://osf.io/za6f2/

## Introduction

As population aging accelerates, older adults constitute the largest subgroup of patients with chronic diseases. Epidemiological data indicate that approximately two-thirds of older adults in high-income countries and 75% of older adults in China live with 1 or more chronic diseases [1-3]. These illnesses require long-term pharmacological management that heavily relies on patient-led self-care outside formal health care settings [1]. Improving medication adherence among older adults is therefore essential to safeguard therapeutic effectiveness and favorable long-term prognosis. However, older adults face multiple barriers, including complex regimens, polypharmacy, cognitive decline, and limited health literacy [4], which frequently lead to medication errors, missed doses, or treatment discontinuation. Suboptimal out-of-hospital medication adherence negatively impacts clinical outcomes, highlighting the urgent need to strengthen self-managed medication behaviors for older adults [4].

Conventional approaches such as hospital-discharge health education deliver only modest benefits [5], and studies further demonstrate that even structured telephone follow-up yields modest and unsustainable adherence gains [4]. In recent years, digital health technologies (DHTs), including electronic monitoring devices, mobile apps, and AI-enabled interactive tools, have emerged as promising solutions to support medication adherence [6-8]. DHT implementations now span diverse care contexts, ranging from hospital inpatient wards to community clinics, long-term care facilities, and rehabilitation services. Even so, age-related cognitive, visual, and auditory impairments combined with limited digital literacy create well-documented implementation barriers for older adults [9], manifesting as difficulties with tool operation, low willingness to engage, and poor real-world performance. Importantly, this digital divide phenomenon cannot be attributed to chronological age alone; it arises from interacting social determinants encompassing educational attainment, geographic location such as urban-rural disparities, and socioeconomic status [10]. Failure to address these structural drivers risks marginalizing older adults from digital health innovations and perpetuating persistent gaps in health literacy promotion.

Multiple existing systematic reviews and meta-analyses have evaluated DHT-supported medication adherence interventions focused on particular chronic disease subgroups. For instance, Miao et al [11] reported beneficial effects of eHealth interventions targeting cardiovascular disease populations; Prawesti et al [12] observed mixed and modest pooled effects of mobile health (mHealth) self-management interventions for patients with chronic obstructive pulmonary disease. Despite these valuable syntheses, prior reviews exhibit notable limitations. Most publications primarily pool and interpret aggregated intervention effect sizes. Few systematically synthesize cross-study shared influencing factors that represent consistent predictors of DHT acceptance and engagement observable across different DHTs and chronic disease groups. Moreover, existing reviews rarely generate concrete, actionable guidance for age-appropriate DHT design and optimization. As a consequence, the field lacks theory-grounded explanatory frameworks to understand heterogeneous intervention outcomes, as well as empirically derived design recommendations built upon real-world older-adult user experiences.

Against this backdrop, revised digital health management strategies are required to mitigate age-related digital health inequities. Although this review is undertaken from a nursing-centered perspective, its outputs hold relevance for multidisciplinary stakeholders, including physicians, pharmacists, and health informaticians, who increasingly participate in deploying digital health resources and delivering medication-adherence support. Accordingly, this scoping review aimed to: (1) identify and characterize DHT interventions for improving medication adherence among older adults, (2) identify and categorize shared influencing factors, and (3) derive actionable age-friendly design principles by mapping these factors to corresponding design solutions, ensuring the framework is derived from the included studies. The following research questions were formulated: first, based on existing studies, what factors influence the use of digital management by older adults with chronic diseases? Second, what key features should age-friendly design and optimization include in digital management systems aimed at enhancing medication adherence among older adults with chronic diseases?

## Methods

### Protocol and Registration

This scoping review was conducted in accordance with the 5-stage framework established by Arksey and O’Malley [13], which involves (1) identifying research questions; (2) identifying relevant studies; (3) selecting studies; (4) charting data; and (5) collating, summarizing, and reporting results, further refined by the Joanna Briggs Institute (JBI) Manual for Evidence Synthesis [14]. The protocol was prospectively registered on the Open Science Framework (DOI: 10.17605/OSF.IO/ZA6F2). Reporting followed the PRISMA (Preferred Reporting Items for Systematic Reviews and Meta-Analyses) flow diagram [15] and the PRISMA-ScR (Preferred Reporting Items for Systematic Reviews and Meta-Analyses Extension for Scoping Reviews) checklist (Multimedia Appendix 1 [16]).

### Eligibility Criteria

Studies were included based on the population, concept, and context (PCC) framework [14]: the *population* was defined as older adults (aged ≥60 years), and studies with mixed-age samples were included only if subgroup data for older adults were separately reported; the *concept* was defined as any DHT, defined as an electronic tool (eg, SMS, mobile app, sensor, smart pillbox, or telepharmacy platform) used to support medication adherence in chronic disease management, and adherence could be measured via validated self-report scales, objective monitoring, or clinical judgment; and the *context* was defined as any health care setting without geographic restrictions. We included original research (cross-sectional, longitudinal, interventional, qualitative, and mixed methods) and secondary studies (systematic reviews and meta-analyses) published in English or Chinese, with full-text availability. Exclusions comprised only abstracts, protocols, letters, unpublished articles, studies not involving chronic diseases, or studies not involving medication. Studies not specifically focusing on older adults or adherence were excluded unless subgroup results for these populations were reported separately.

### Information Sources

The literature search was performed in 2 phases. The initial search covered January 1, 2013, to September 8, 2025, with a subsequent update conducted on September 2, 2026, covering September 9, 2025, to August 2026. Seven databases were searched: MEDLINE (via PubMed), Web of Science, CINAHL (via EBSCOhost), SinoMed, CNKI, Wanfang, and VIP. The selection was justified by their complementary coverage: MEDLINE for broad biomedical literature; Web of Science for multidisciplinary technology and aging research; CINAHL for nursing and allied health perspectives; and the 4 Chinese databases for capturing Chinese-language evidence given China’s large aging population and rapid DHT implementation. To minimize the risk of missing studies, we performed both backward citation chasing (reviewing reference lists of included studies) and forward citation chasing (using Google Scholar to identify citing articles).

### Search

The search strategy combined 3 concept groups using MeSH terms, field modifiers (eg, title [TI] and abstract [AB]), and a wide range of synonyms: (1) older adult terms, (2) DHT intervention terms, and (3) medication adherence and chronic disease terms. The complete strategies for all databases, copied exactly as run, are provided in Multimedia Appendix 2. The number of records retrieved per database and the duplication process are reported in Multimedia Appendix 2 and the PRISMA flow diagram.

### Selection of Sources of Evidence

All retrieved records were imported into EndNote 2021 (Clarivate) for the literature screening process. Two reviewers (LL and LZ) independently screened titles and abstracts against the eligibility criteria. Potentially relevant full-text articles were then independently assessed by the same 2 reviewers. Discrepancies at any stage were resolved through discussion, with arbitration by a senior reviewer (ZC) when consensus could not be reached. Reasons for exclusion during the full-text review were documented in the PRISMA-ScR flow diagram, which summarizes the screening process and ensures traceability of the decision-making process. Interrater reliability was not formally calculated, consistent with scoping review practice [17].

### Data Charting Process

Prior to analyzing the final included studies, we developed a data extraction form through team discussion, updates, optimizations, and resolution of all differences, which included author, publication year, study purpose, population characteristics, tools for assessing medication adherence, and key findings. Given the large number of finally included studies, the full table is presented in Multimedia Appendix 3. In addition, to provide a theoretical lens for organizing and interpreting the heterogeneous extracted data, we drew on 2 complementary theories: social cognitive theory (SCT) [18] and person-environment fit (P-E fit) theory [19]. SCT posits that personal, behavioral, and environmental factors interact bidirectionally, making it suitable for understanding how older adults engage with digital health tools. P-E fit theory emphasizes that behavioral outcomes depend on the congruence between individual capabilities and environmental demands, which is particularly relevant for explaining why some older users adopt DHTs while others abandon them. Prior to data extraction, we operationalized these theoretical constructs into a structured coding manual to guide data charting. The manual specified 4 a priori categories: individual-level factors (derived from SCT’s personal factors), technical-level factors (derived from P-E fit theory’s environmental conditions), social-environmental factors (derived from SCT’s environmental factors), and interventional factors (which emerged inductively during pilot coding). The coding manual was pilot-tested on 5 randomly selected studies, refined through team discussion, and then applied to all included studies by 2 independent reviewers (LL and LZ). Discrepancies were resolved by consensus or senior reviewer arbitration. We did not contact study authors for missing information, as this is not mandatory for scoping reviews and is noted as a limitation.

### Data Items

To ensure the richness and completeness of the final results, we extracted the following variables from each included study using Microsoft Excel 2021: author, publication year, study design, sample characteristics (including age, gender, living situation, and number of medications), DHT type, medication adherence assessment method, medication use stages addressed, usability metrics (if reported), key findings related to adherence, and reported barriers and facilitators. These data were used to generate visual maps of the studies rather than to serve as the final data presentation.

### Synthesis of Results

To synthesize the heterogeneous findings extracted from the included studies, we adopted a multistep analytical process that progressively moved from descriptive mapping to explanatory modeling and finally to translational framework development. This process was guided by the same 2 theories (SCT and P-E fit) described above. In the first stage, descriptive mapping, we aimed to objectively characterize the distribution of existing research and identify structural gaps. To operationalize this, we drew on the behavioral dimension of SCT and disaggregated the broad behavior of medication management into 5 sequential and observable stages (prescription, pickup, storage, daily routine, and refill). By mapping the included studies onto these stages, we assessed which phases of the medication behavior chain have received research attention and which remain understudied. This stage yielded a study distribution bubble chart and a summary of underrepresented dimensions, moving beyond a general assessment of adherence to a more granular, behavior-focused visualization of the research landscape.

In the second stage, explanatory modeling, we sought to move from describing the research landscape to explaining why intervention outcomes vary across studies. Using the coded data generated from the SCT- and P-E fit theory–informed manual, we aggregated all reported barriers and facilitators from each study under the 4 coding categories. Through an iterative process of comparison and abstraction, we consolidated them into a 4-level influencing factor model. This model elucidates how mismatches across these levels, such as high technical complexity with low digital literacy, and matches, such as when strong social support features in DHT design align with the needs of older adults living alone, explain the heterogeneous intervention outcomes observed in the literature. This stage thereby provided a mechanistic understanding of intervention performance beyond mere effect aggregation.

In the third stage, translational framework development, we aimed to convert the mechanistic understanding derived from the second stage into actionable guidance for DHT design and optimization. To operationalize this, we applied P-E fit theory’s match logic to the user-reported usability barriers extracted from the coded data. Through an inductive thematic synthesis, we grouped these empirical findings into 5 distinct age-friendly design dimensions: perceptibility, comprehensibility, operability, emotional warmth, and security-interoperability. This framework was not imposed from existing guidelines but was directly derived from the real-world experiences of older adults. To enhance its practical utility, we further established a mapping matrix that explicitly links each user-reported barrier to its corresponding influencing factor level and its derived design solution. This mapping demonstrates how the theoretical understanding of barriers directly translates into actionable design guidance, bridging the gap between explanatory theory and practical optimization. The complete theoretical-analytical workflow is visualized in Figure 1.

**Figure 1:**
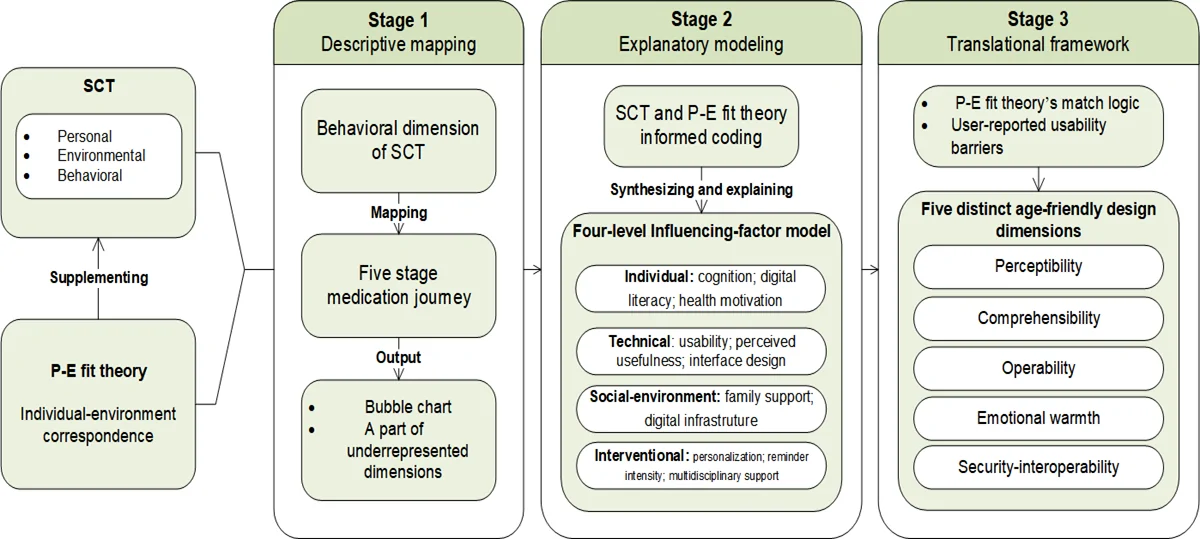
Theoretical-analytical workflow. P-E fit: person-environment fit; SCT: social cognitive theory.

## Results

### Selection of Sources of Evidence

The study selection process is presented in Figure 2. The initial search was performed on September 8, 2025, covering the period from January 1, 2013, to September 8, 2025. A total of 10,393 records were identified through database searching. After removing 726 duplicates, 9667 records remained. Two reviewers independently screened titles and abstracts, resulting in the exclusion of 8924 records. The remaining 743 records underwent full-text assessment, of which 32 were unavailable and 661 were excluded for not meeting the eligibility criteria. Backward and forward citation chasing of the 50 included studies identified no additional eligible records. A total of 50 studies were included in the initial review.

**Figure 2:**
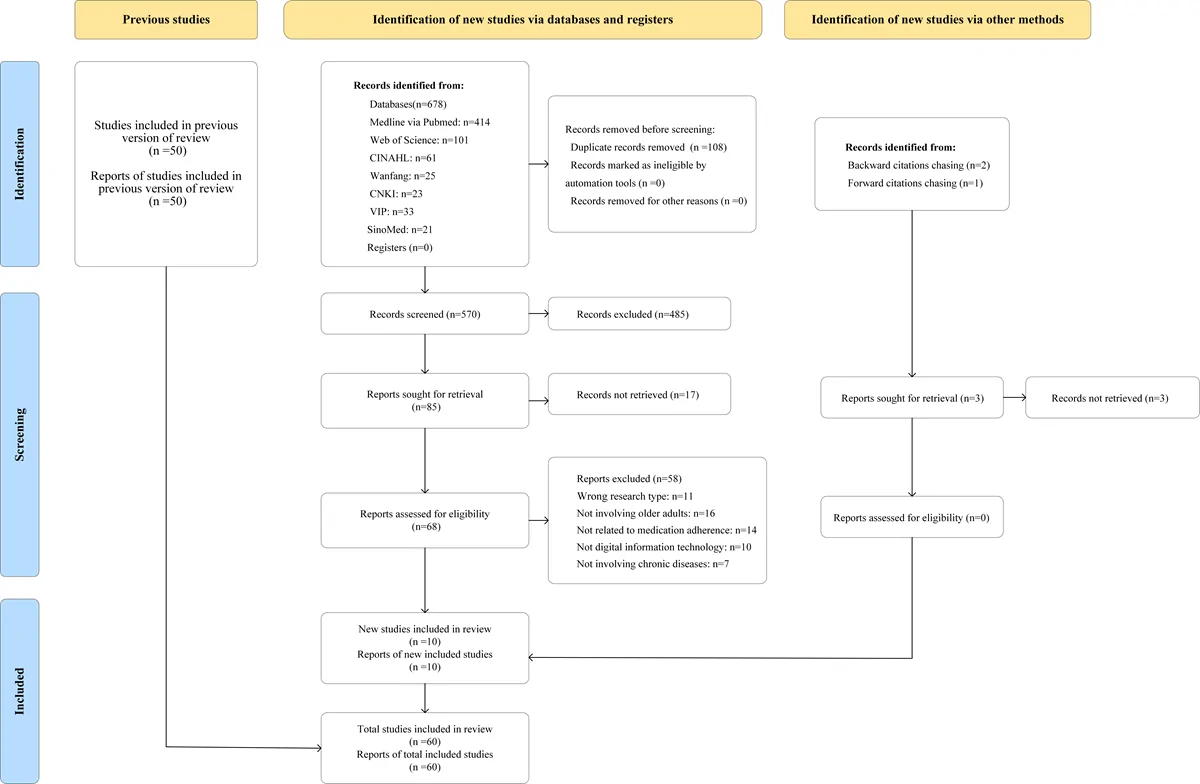
PRISMA-ScR (Preferred Reporting Items for Systematic Reviews and Meta-Analyses Extension for Scoping Reviews) flowchart for study selection.

An updated search was conducted on September 2, 2026, covering the period from September 9, 2025, to August 2026, using the same databases and search strategies. This update identified 678 records. After removing 108 duplicates, 570 records were screened. Of these, 485 were excluded at the title and abstract stage. The remaining 85 full-text articles were assessed for eligibility, of which 17 were unavailable and 58 were excluded. Backward and forward citation chasing of the newly identified studies yielded no additional eligible records. Ten new studies met the inclusion criteria. Combining both searches, a total of 60 studies were included in this scoping review [11,12,20-77].

### Characteristics of Sources of Evidence

#### Overview of Included Studies

To facilitate comprehensive reporting, the detailed characteristics of all 60 studies included from both searches are presented in Multimedia Appendix 3. Of these, 21 were randomized controlled trials, 19 were secondary studies (including systematic reviews and meta-analyses), and the remaining 20 used quasi-experimental, qualitative, or other methodological designs. As shown in Figure 3, research output in this field remained relatively sparse between 2013 and 2020, with a marked acceleration observed from 2022 onward. Overall, 46 (76.7%) studies were published between 2022 and August 2026, reflecting rapidly growing research interest in digital health technology-supported medication adherence management among older populations. All included studies fully or partially involved older adults, with a slight male predominance, particularly pronounced in cardiovascular disease studies. Polypharmacy (averaging 4-6 medications) and low-to-moderate medication adherence were commonly reported across studies. However, reporting of socially vulnerable subgroups was notably inconsistent and insufficient across the study base. Only 1 study specifically recruited older adults living alone [25], 2 studies focused on older adults in low-resource settings [57,72], and only 1 study published in 2026 specifically included older adults with frailty or cognitive impairment [75].

**Figure 3:**
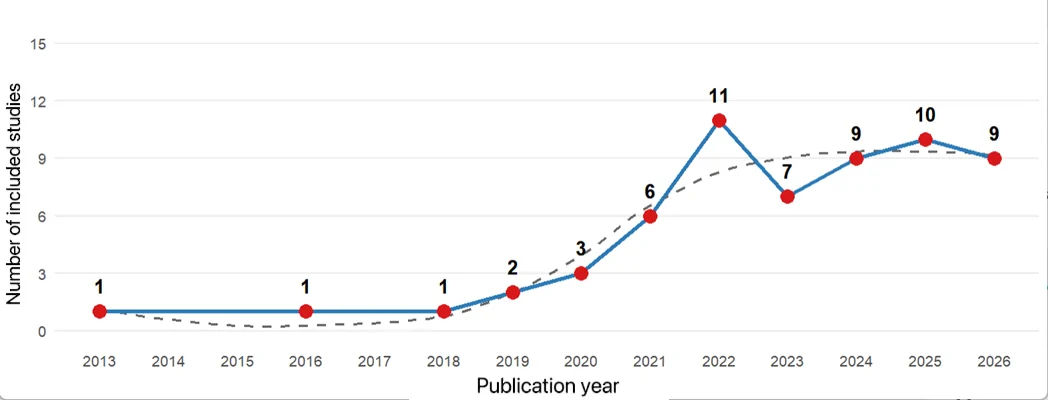
Distribution of included studies by publication year (N=60).

#### Classification and Distribution of DHT Interventions

Based on the depth of user interaction and technological maturity, all included DHT interventions were categorized into 5 groups via inductive coding: (1) conventional and basic media (eg, SMS and telephone calls), (2) interactive digital tools (eg, mobile apps and cloud-based platforms), (3) interpersonal support strategies (eg, WeChat-based care and telepharmacy services), (4) context-aware intelligent devices (eg, smart pillboxes and sensor-based monitoring systems), and (5) unspecified or mixed interventions. However, the fifth category was not included in the analysis and was only presented as data. The final report was based on the clear classification of the previous 4 categories. The distribution of DHT categories across 2 publication periods (2013-2021 and 2022-2026) is presented in Figure 4. Interactive digital tools accounted for the largest proportion, whereas context-aware intelligent environment studies were the least frequent. Except for conventional and basic media interventions, all DHT categories showed increased publication counts in the 2022-2026 period relative to the earlier period, reflecting a transition from unidirectional notification tools toward bidirectional interaction and context-aware intervention designs.

**Figure 4:**
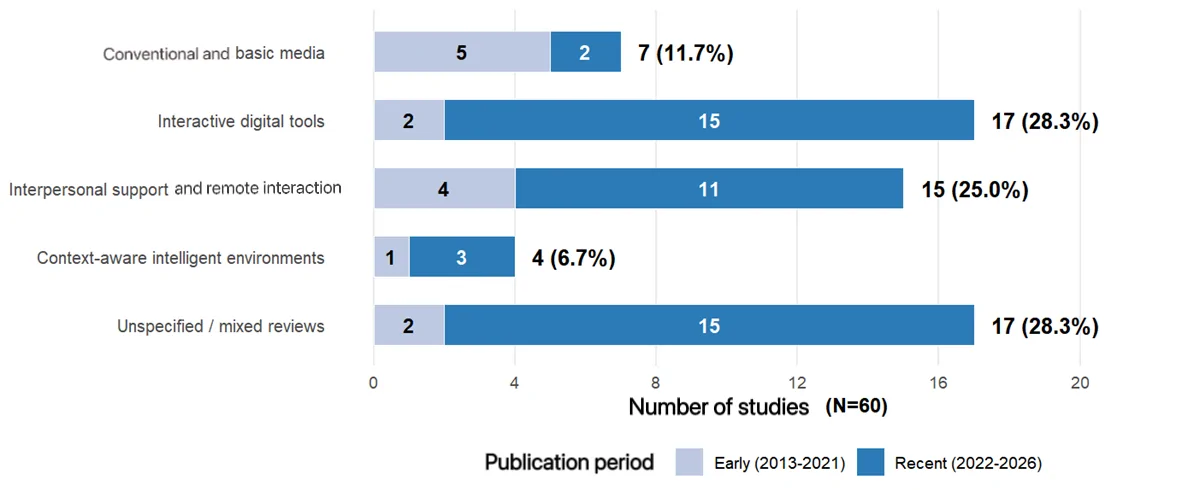
Distribution of studies by digital health technology (DHT) category and publication window.

#### Medication Journey

The completed medication journey includes prescription, pickup, storage, daily routine, and refill [78]. To visualize the temporal evolution and distribution of DHT research across the 5-stage medication journey, we constructed a study map (Figure 5), with publication year on the x-axis, medication journey on the y-axis, bubble size representing study count, and color indicating DHT type (based on the main DHT mentioned). The distribution of studies across the medication journey showed a marked concentration on the daily routine stage, which dominated the entire study period from 2013 to 2026. All 4 DHT categories were represented at this stage, with research activity increasing after 2021. Interventions based on interactive digital tools and interpersonal support with remote interaction constituted the largest cluster. In contrast, the remaining 4 stages, including prescription, pickup, storage, and refill, remained critically underexplored, with only sporadic studies addressing each. Temporal disparities were also evident. Research on daily routine behaviors dated back to 2013 and accumulated steadily over time, whereas investigations targeting other stages did not emerge until 2020. By technology type, conventional and basic media interventions were limited to the daily routine and prescription stages, with no studies identified for pickup, storage, or refill. Context-aware intelligent devices were only applied in the daily routine and storage. Notably, no single DHT type comprehensively covered all stages of the medication journey.

**Figure 5:**
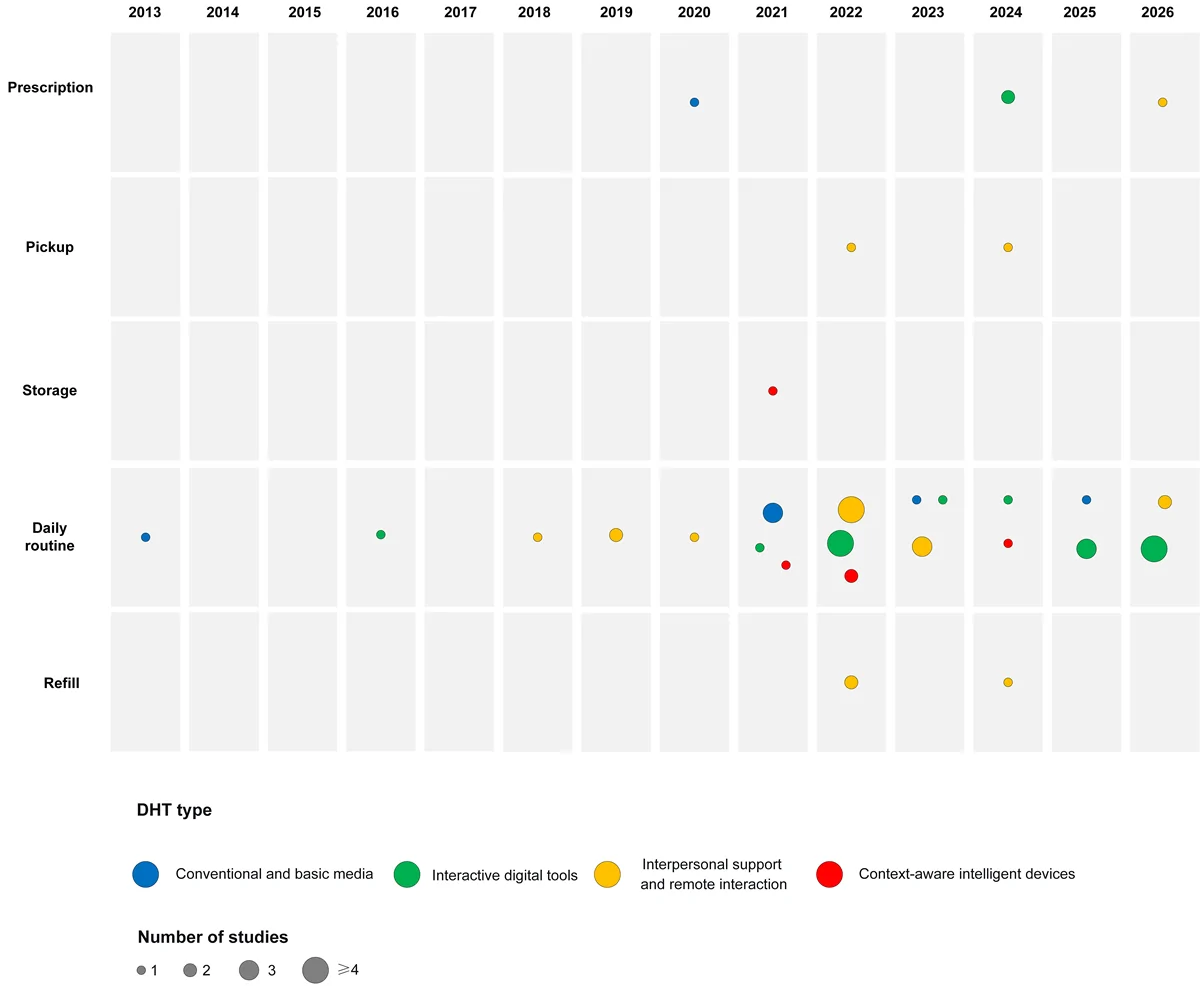
Distribution of digital health technologies across the medication journey in older adults with chronic diseases (2013-2026).

#### Approaches to Measuring Medication Adherence and Usability

Considerable heterogeneity existed regarding methods used to assess medication adherence. A total of 28.3% (17/60) of included studies did not explicitly report the adherence measurement instrument. Among studies that specified assessment approaches, the Morisky-8 Scale (16/60, 26.7%) was most frequently applied, followed by others (15/60, 25%), self-developed questionnaires (6/60, 10%), and objective monitoring data (6/60, 10%). Regarding usability evaluation, only 1 study conducted a formal standardized assessment using validated scales such as the Usability Metric for User Experience (UMUX) [30]. A few usability-related findings were derived exclusively from qualitative participant feedback, lacking objective, quantitative usability data [25,32,35,38].

#### Effect Observations and Underrepresented Areas

Consistent with standard scoping review methodology, formal risk of bias assessment was not performed in this review. All effect-related findings are presented as descriptive observations, and no causal inferences are drawn. Among the controlled intervention studies included, 6 reported nonsignificant between-group differences, indicating no measurable improvement in medication adherence after DHT-based interventions [37,43-45,52,53]. These interventions with nonsignificant effects shared several common contextual features: most adopted unidirectional SMS or telephone reminders with limited interactivity, used nonvalidated adherence assessment instruments, and recruited general community-dwelling older adults without tailored support for socially vulnerable subgroups. These features represent observed co-occurring patterns rather than confirmed causal contributors to nonsignificant outcomes. Table 1 consolidates these patterns and other under-represented dimensions identified across all 60 included studies, with interpretive notes for each dimension indicating its potential relevance to the observed variability in intervention effects.

**Table 1:** Summary of underrepresented dimensions (N=60 studies; detailed sample sizes for each included study are available in Multimedia Appendix 3.

Dimension and underrepresented aspect			Supporting studies, n (%)		Interpretive note
DHT^a^ type					
	Unidirectional basic media reminders (SMS and telephone)	7 (11.7)		Predominant in several nonsignificant studies; may lack sufficient engagement to sustain adherence	
	Context-aware intelligent environments	4 (6.7)		Limited data precludes assessment of potential effectiveness	
Population subgroups					
	Older adults living alone	1 (1.7)		May require interpersonal support beyond automated reminders	
	Low-resource rural settings	2 (3.3)		Infrastructure barriers may limit sustained DHT use	
	Frail or cognitively impaired older adults	1 (1.7)		Targeted usability and support needs have not been fully examined	
Medication journey					Underrepresented across the entire study base; may represent missed opportunities for adherence support
	Prescription	4 (6.7)			
	Refill	3 (5.0)			
	Pickup	2 (3.3)			
	Storage	1 (1.7)			
Adherence measurement					
	Unspecified assessment methods	17 (28.3)		Limits comparability of adherence outcomes across studies	
Usability evaluation					
	No usability assessment reported	49 (81.7)		May impede sustained engagement, particularly among older users with low digital literacy	
	Nonstandard usability assessment	10 (16.7)		May limit generalizability	
	Formal usability metrics reported	1 (1.7)		—^b^	
Clinical integration					
	Bidirectional EHR^c^ interoperability assessed	None		May limit perceived usefulness and integration into care	
Follow-up duration					
	Long-term outcome assessment (>12 months)	Uncommon		Cannot capture sustained adherence effects	

^a^DHT: digital health technology.

^b^EHR: electronic health record.

^c^Not applicable.

### Factors Influencing Digital Interventions

Integrating SCT [18] and P-E fit theory [19], factors shaping older adults’ engagement with DHT-supported medication management were synthesized inductively into a 4-level framework. This framework offers a theory-grounded explanation of the observed variability in intervention effects. The hierarchical structure of this framework is visualized using an interactive sunburst diagram (Multimedia Appendix 4). The inner ring represents the 4 theoretical dimensions derived from the integrated framework, with successive outer rings detailing subfactors and their specific manifestations as reported in the included studies. The proportional size of each sector reflects the relative richness of factors within each category, thereby enabling rapid identification of dimensions and subfactors that are more extensively addressed in the current literature. Detailed descriptions and mechanisms are presented in Table 2.

**Table 2:** Factors influencing digital interventions for older adults.

Categories and key influencing factors		Mechanism of action and manifestations
Individual level		
	Cognition and physical functions	Digital divide: age-related decline of memory, visuospatial skill, expression, and operational capacity creates barriers to operating DHTs^a^.
	Digital literacy and attitude	Digital exclusion due to the unfamiliarity with new technologies, anxiety, concerns about privacy, and preferences for traditional medicine.
	Personal health motivations	Unmotivated: low priority and weak motivation for health management.
Technical level		
	Usability	Intuitive interfaces and low learning-curve requirements improve sustained user engagement. Cumbersome operations increase abandonment risk.
	Usefulness	Users are more likely to continue usage when they subjectively perceive the tool contributes to better medication self-management.
	Design for older adults	Features including large fonts, high-contrast colors, and voice interaction mitigate age-related functional limitations; defective voice recognition or unresponsive touch controls trigger frustration and dropout.
Social-environment level		
	Social support	Encouragement from family, friends, and health care providers (increased confidence and device adoption); for older adults living alone, impersonal DHTs consistently fail to engage them.
	Digital infrastructure and accessibility	Network coverage and affordable equipment are the basic requirements (necessary for sustained use).
Intervention program level		
	Alignment with patient preferences	Intervention content and delivery modes matching user preferences improve acceptance.
	Personalization and interactivity	Personalized feedback (improved adherence); interactivity (sustained engagement).
	Usage intensity and support	Joint interventions by multiple technical teams; information overload (resistance and reduced adherence).

^a^DHT: digital health technology.

At the individual level, cognitive and physical functioning [23], digital literacy and attitudes [64], and personal health motivation [45] consistently shaped older adults’ ability and willingness to engage with DHTs. Of note, higher educational attainment predicted greater digital engagement, whereas negative attitudes, including digital anxiety and privacy concerns, diminished adoption. At the technical level, usability [32], perceived usefulness [40], and age-friendly design [33] features shaped user experiences. Age-friendly design elements such as large-font display and voice-enabled interaction were associated with improved usability, whereas unresponsive interfaces and poor voice recognition contributed to user frustration and disengagement. At the social-environmental level, social support from family and providers boosted confidence and sustained engagement [42]. Older adults living alone and lacking such interpersonal support faced particular barriers when interacting with fully automated, impersonal digital tools [25]. Digital infrastructure, network access, and device affordability acted as necessary preconditions for implementation, especially within low-resource contexts [57]. At the intervention program level, alignment with patient preferences [52], personalization, interactivity [61], and multidisciplinary support [31] were associated with superior outcomes. However, high-intensity reminders improved short-term adherence but caused long-term resistance [67], and excessive reminder frequency led to alert fatigue and intentional non-adherence [42].

### Synthesis of Age-Friendly Design Dimensions Derived From Included Studies

Based on thematic synthesis of usability-related barriers and corresponding optimization strategies extracted from included publications, 5 empirically grounded age-friendly design dimensions were generated: perceptibility, comprehensibility, operability, emotional warmth, and security-interoperability. Perceptibility addresses age-related visual, auditory, and memory-related limitations through multimodal reminder functions and high-contrast interface settings [30]. Comprehensibility targets barriers originating from insufficient digital literacy by simplifying information presentation and lowering learning burdens [33]. Operability focuses on physical interaction requirements, including enlarged touch targets and adaptive interface logic [11]. Emotional-warmth-oriented functions respond to motivational deficits and lack of caregiver support by incorporating positive reinforcement and family-linked communication channels [24], particularly critical for older adults living alone [25]. The security-interoperability dimension encompasses data protection requirements as well as technical demands for bidirectional connectivity between DHT tools and clinical EHR workflows [79]. Mapping between user-reported barriers and corresponding age-friendly design solutions is summarized in Table 3.

**Table 3:** Mapping influencing factors to age-friendly design dimensions.

Design dimension and specific barrier			Influencing factor level		Design solution
Perceptibility					
	Age-related visual/auditory decline and forgetfulness	Individual		Multimodal reminders (visual, auditory, and tactile), large fonts, high-contrast colors, and voice broadcasts.	
Comprehensibility					
	Low digital literacy and technology anxiety	Individual		Use simple, clear interfaces, videos, images, and skeuomorphic design (eg, a “virtual pillbox”) to reduce the learning curve.	
	Information overload and alert fatigue	Interventional		Prioritize essential information, allow user-controlled reminder schedules, and transparent data handling.	
Operability					
	Complex navigation and small touch targets	Technical		Large touch targets, gesture tolerance, voice commands, and adaptive interface that simplifies with use.	
Emotional warmth					
	Weak health motivation	Individual		Positive reinforcement messages and personalized encouragement (eg, “Great job this week!”).	
	Lack of family/peer support	Social-environmental		Family/caregiver communication channels and peer group features.	
	Living alone	Social-environmental		Incorporate regular human-like check-ins and avoid purely automated, impersonal systems.	
Security-interoperability					
	No integration with clinical workflows	Technical and interventional		Bidirectional EHR^a^ integration, seamless clinician feedback loops, and strengthened information security.	

^a^EHR: electronic health record.

## Discussion

### Summary of Evidence

This scoping review synthesized applications of DHTs targeting medication adherence among older adults with chronic diseases, with 60 studies ultimately included. Research activity has grown rapidly since 2022, reflecting markedly accelerated global academic interest in digitally enabled medication management for older adults. Synthesizing all results, this review yielded 3 progressive tiers of analytical outputs. The descriptive tier mapped DHT typologies and research distribution across the full medication journey. The explanatory tier constructed a 4-level influencing factor framework to identify conditions under which DHTs succeed or fail among older adults. The translational tier derived 5 age-friendly design dimensions based on real-world usability barriers reported by older adults. This multitiered analysis addresses a long-standing deficiency in this field: numerous randomized controlled trials report mixed intervention outcomes, yet few systematically unpack the underlying drivers of intervention failure and provide optimization solutions.

### Comparison With Previous Reviews: Limitations and Contributions

#### Overview

Prior studies have largely adopted review approaches to examine whether DHTs produce beneficial effects. For instance, Kim et al [80] demonstrated significant improvements in medication adherence following DHT interventions across 5174 participants. By contrast, Thuy et al [81] synthesized 15 primary studies and found limited, inconsistent evidence for adherence improvements delivered by reminder-based DHTs among community-dwelling older adults. While existing reviews confirm the potential of DHTs to enhance medication adherence, they repeatedly document inconsistent evidence and mostly stop at descriptive observations [82]. Three key limitations persist within this body of literature. At the theoretical-interpretive level, reviews conduct only statistical tests of heterogeneity without deeper mechanistic analyses. Methodologically, they overlook the medication journey construct and lack systematic usability assessment, which prevents identification of structural gaps in research resource allocation. From a system-integration perspective, insufficient attention is paid to the enrolment of vulnerable subgroups and clinical interoperability, undermining the fairness and ecological validity of research findings. This scoping review advances existing knowledge along the following 3 dimensions.

#### Extending From Effect Aggregation to Mechanistic Understanding

Previous systematic reviews typically limit heterogeneity investigations to statistical surfaces, attributing between-study variance to methodological features such as follow-up duration, adherence measurement instruments, or disease categories [83]. Theory-driven moderators and mechanistic explanatory frameworks are rarely incorporated. Such statistics-oriented approaches can describe the existence of discrepancies but cannot illuminate why discrepancies emerge. A key distinction of the present work is that it does not merely restate the well-established conclusion that DHTs yield mixed effects. By integrating SCT [18] and P-E fit theory [19], this multifactor analytical framework treats heterogeneity itself as a core research object. It explains how matches and mismatches among individual capacities, age-friendly technical attributes, external social support, and intervention characteristics generate divergent DHT intervention outcomes. It further theoretically unpacks root sources of heterogeneity and addresses unresolved scientific questions left by earlier reviews.

#### Methodological Expansion: Medication Journey and Usability Evaluation

One critical contribution of this review lies in its systematic mapping of evidence across the complete medication journey. Prior literature acknowledges medication management as a multistage process, and Lunghi et al [84] proposed a 3-step framework comprising prescription, patient acceptance, and ongoing monitoring. The Maidment research group also conceptualized medication management among older adults as a complex 5-stage process [85]. Nevertheless, no prior review has systematically quantified the distribution of DHT-related research across these stages.

Our study map reveals pronounced imbalance. Research focusing on the daily routine has accumulated steadily since 2013, whereas studies covering the remaining 4 stages did not emerge until 2020 and remain severely underinvestigated to date. This imbalance can be partially interpreted through cognitive attribution and technical convenience perspectives. However, real-world nonadherence stems from far more complicated sources, including prescription errors, failure to pick up, improper storage, and delayed prescription refills [86]. Previous epidemiological work indicates that 14.6% to 20% of patients with chronic conditions never initiate treatment after receiving a prescription, meaning many patients disengage well before reaching the daily-dosing phase [87,88]. Furthermore, no single category of DHT comprehensively covers all of the medication journey. This offers a potential explanation: interventions targeting only one segment of the medication journey leave other unaddressed barriers intact and may therefore attenuate overall intervention benefits. By quantifying uneven stage-level coverage, this review points future research toward a shift from single-point reminder interventions toward full-chain medication journey support.

Regarding usability evaluation, only 1 included study deployed validated standardized usability scales [30]; most other studies relied solely on qualitative participant feedback or omitted usability assessment entirely. This finding highlights a systematically underquantified problem within DHT-enabled medication adherence research. This reporting gap carries multiple consequences. First, when interventions fail to deliver expected benefits, researchers struggle to distinguish whether poor performance originates from ineffective intervention content or suboptimal interface design [89]. Second, the absence of baseline usability benchmarks impedes cross-product comparative evaluation [90]. Third, without standardized usability assessment, the field cannot systematically accumulate replicable knowledge regarding which specific design elements work for older adults [91]. While qualitative feedback yields rich user insights, its fragmented nature hinders development of testable, reproducible evidence bases for subsequent research. Finally, clinical decision-making lacks explicit usability evidence, which may block effective DHTs from real-world implementation [89].

Addressing these gaps, this review characterizes the magnitude of missing standardized usability assessment and provides a baseline reference for future investigators. Prior publications have confirmed that standardized usability instruments are applicable for older adults [30], yet this review demonstrates that such tools are rarely deployed within this research domain, indicating a notable methodological gap.

#### Expanding System Integration Perspectives: Vulnerable Subgroups and Interoperability

Representation of socially vulnerable older subgroups was extremely limited across included studies (Table 1). Only 1 study specifically recruited older adults living alone [25]; 2 studies investigated older adults within low-resource settings [57,72], and 1 study targeted older adults who were frail or cognitively impaired [75]. While some earlier publications examine certain vulnerable subgroups, they concurrently acknowledge sparse evidence for these populations [92]. Moreover, most existing studies analyze older adults as a homogeneous collective and lack unified framework comparative analyses across socially vulnerable older subgroups [93]. This structural imbalance in study recruitment produces 2 adverse consequences. First, socially vulnerable older individuals who stand to benefit substantially from DHT interventions are systematically undersampled, further amplifying intra–age-group digital health divides and health inequities. Second, DHT interventions optimized for generally healthy older adults often fail to accommodate unique health requirements and real-world contexts of vulnerable subgroups. Future research should deliberately enroll these understudied vulnerable populations and develop tailored interventions built upon subgroup-specific pain points.

With respect to interoperability, none of the included studies explicitly evaluated integration with electronic health records or established clinical workflows. Earlier works report one-way data transmission toward clinicians [79], but overlook interoperability as a foundational enabler for closed-loop clinical integration. When decoupled from clinical workflows, medication-use data generated by DHTs cannot feed into clinical decision-making cycles. Clinicians cannot adjust prescriptions based on such data nor verify data accuracy during routine follow-ups [94]. Such data silos confine DHTs to research-only instruments and prevent their translation into routine clinical care pathways [94]. Accordingly, future work should promote interdisciplinary collaborative research, explore secure technical integration solutions, and evaluate tangible improvements to clinical decision-making and patient-centered outcomes.

### Four-Level Influencing-Factor Model and Practical Translation

DHTs create new opportunities for out-of-hospital health management, yet heterogeneous intervention outcomes depend on preconditions concerning capacity, willingness, and sustained usage among older adults [79]. The 4-level model developed in this review, built upon SCT and P-E fit theory, systematically explains interactive mechanisms operating across these 4 domains. As shown in the sunburst diagram (Multimedia Appendix 4), the individual level occupies the largest sector area, indicating that it has received the most extensive research attention, whereas the social-environmental level shows the smallest sector area, reflecting that existing literature on social support and infrastructure-related factors remains markedly insufficient. The following will elaborate factor definitions and derived practical implementation strategies for each level.

At the individual level, age-related cognitive decline directly shapes older adults’ processing of reminder messages, retention of operational procedures, and speed of interpreting interface feedback. Cognitive and physical functional decline and digital literacy currently represent the primary research focuses (Multimedia Appendix 4). When cognitive load exceeds individual capacities, even simple SMS reminders may be ignored or misinterpreted. Digital literacy governs older adults’ adaptation speed and self-troubleshooting capacity when encountering novel technologies [95]. Health motivation reflects intrinsic drive to overcome initial learning curves [96]. Although it is critical for maintaining long-term medication behaviors, research on this factor remains relatively sparse (Multimedia Appendix 4). These 3 factors frequently exert cumulative effects that jointly shape older adults’ DHT adoption capacity and willingness to engage. Therefore, for clinical practice, nurses should complete 3 baseline assessments prior to DHT implementation. Cognitive screening can be performed using brief instruments such as the Mini-Cog [97]. Simplified digital-literacy scales can measure digital self-efficacy [98]. Open-ended questioning can be applied to gauge health motivation levels. Patients can then be stratified into 4 profiles with corresponding implementation pathways. Fully featured interactive applications may be directly recommended for participants with high digital literacy and high motivation. Individuals with low literacy but high motivation require hands-on training alongside concise graphic guidance. Motivational interviewing should be prioritized for patients with high literacy but low motivation. Those with both low literacy and low motivation may begin with basic 1-way SMS reminders before gradual functional escalation.

At the technical level, usability determines whether older adults can complete tasks under acceptable cognitive-load burdens. Intuitive interfaces, unambiguous feedback, and low learning-curve requirements constitute prerequisites for sustained engagement [99]. Perceived usefulness shapes user valuation of tool utility and is theoretically a key factor in promoting continued use, but empirical research in this area remains limited (Multimedia Appendix 4). Age-friendly design features account for a prominent proportion of entries (Multimedia Appendix 4), indicating that interface adaptation is a current research priority, primarily reflected in whether interface components and interaction modalities align with older adults’ sensory and motor capabilities. In clinical practice, usability should serve as a prerequisite for technical adoption [100]. Nursing teams should lead clinical pretrials for DHT usability. For example, representative older participants can be recruited for 1-week real-world field testing, with outcome metrics including task error rates, task-completion times, System Usability Scale scores, and subjective difficulty feedback.

At the social-environmental level, encouragement and practical assistance from family members and clinical providers substantially mitigate user frustration, while digital-infrastructure access forms fundamental usage prerequisites. Multimedia Appendix 4 reveals that both social support and infrastructure factors are under-researched relative to their real-world importance. The scarcity of studies on living alone status and infrastructure is particularly noteworthy, as these 2 factors have a decisive influence on a considerable proportion of older adults. For instance, older adults living alone exhibit significantly higher dropout risk owing to the absence of routine interpersonal support. In clinical practice, living-alone status should act as a core stratifying variable for DHT selection. Solutions embedding human-interaction modules ought to be prioritized for this subgroup [101]. Digital-infrastructure assessments should also be incorporated into patient profiles as constraining criteria for technology recommendations.

At the intervention program level, personalization determines alignment between intervention content and individual medication regimens and daily rhythms. Interactivity supports long-term participant engagement, and reminder intensity requires careful balancing [102]. Multimedia Appendix 4 shows a relatively balanced distribution of factors at this level, suggesting moderate research attention has been received, though this area has not yet become a research hotspot. Among these, patient preference-related content remains relatively sparse, yet this factor is likely critical for influencing initial acceptance and sustained willingness to use. In practical application, greater attention should be paid to the needs of patients and their usage preferences. Regarding the intensity of reminders, an adaptive-reminder strategy is recommended for real-world deployment. For instance, higher-frequency alerts are deployed initially to support habit formation. Reminder density is then gradually reduced according to observed medication-taking stability, finally transitioning toward on-demand alert triggers.

In summary, the 4-level model should move beyond theoretical description and be translated into standardized assessment checklists and stratified intervention algorithms embedded within clinical nursing pathways. Correspondingly, nurses’ roles evolve from basic technical instructors toward technology-matching decision-makers who empower older adults.

### Implications of the Age-Friendly Design Framework for DHT Development and Evaluation

Each of the 5 design dimensions thematically synthesized within this review is directly grounded in empirically reported barriers articulated by older adult end users. Compared with existing generic age-accessibility guidelines, this framework possesses 2 distinct strengths.

The first strength is situational specificity. The 5 dimensions were not deductively derived from universal design principles. Instead, they were inductively summarized from real-world difficulty narratives of older adults within the concrete context of medication management. Take the emotional warmth dimension as an illustration: this construct rarely appears within generic accessibility principles [103], yet it is repeatedly highlighted within qualitative feedback from older adults living alone and other end users. Medication-taking is inherently long-term and repetitive; purely functional tools lacking emotional support struggle to sustain durable behavior change. The second strength lies in its dynamic closed-loop property. The framework establishes explicit mapping pathways between identified usage barriers and concrete design solutions. Development teams can clearly define targeted problems and corresponding functional responses for every dimension, avoiding common design inertia that adds features without addressing actual user needs. Perceptibility maps to multimodal reminders and visual enhancement, comprehensibility maps to simplified information presentation and reduced learning burden, operability maps to enlarged touch targets and voice-command functionality, emotional warmth maps to positive reinforcement and interpersonal linkage, and security-interoperability maps to data-privacy safeguards and clinical-system integration.

It is important to highlight that interoperability depends on system-level infrastructure. Its realization demands institutional preconditions including development of health-information standards, construction of regional health-information platforms, and formal data-sharing agreements [104]. Even so, interoperability represents only one component of a broader evaluation framework. Other equally vital yet underemphasized metrics include privacy and data security, accessibility (device availability and network coverage), cognitive load, long-term sustainability, and user autonomy. Future evaluations should adopt this multidimensional framework rather than focusing on isolated single indicators.

For clinical appraisal purposes, this framework supplies 5 reference quantifiable evaluation dimensions. When reporting DHT intervention effectiveness, future studies should document achievement status across these 5 dimensions. Examples include verifying whether perceptibility is delivered through multimodal reminders, whether operability meets acceptable thresholds, and whether security-interoperability is achieved through connections to local health-information platforms. Such reporting enhances intervention reproducibility and cross-context comparability. Sustainable DHT deployment for older adults does not rely on feature proliferation or rapid technical iteration. Instead, success hinges on genuine embedding within older adults’ lived contexts, cognitive rhythms, and social-network environments [105]. The nursing discipline occupies a central position in advancing age-friendly digital health innovation. Real-world implementation nevertheless requires full-phase participatory co-design spanning concept through deployment, with involvement from older adults, caregivers, and interdisciplinary teams consisting of physicians, nurses, pharmacists, and health informaticians. This collaborative process guarantees contextual appropriateness and long-term sustainability [106].

### Limitations

This scoping review has several limitations. First, the study did not systematically search gray literature sources, although a few theses were retrieved through the main database searches; this may have introduced reporting bias. Second, cost factors were also important factors affecting digital interventions; however, as this drawback cannot be perfectly addressed from a nursing perspective, no in-depth discussion was conducted. Third, in accordance with PRISMA-S (Preferred Reporting Items for Systematic Reviews and Meta-Analyses Literature Search Extension), the following items were not applicable to the methodology of this scoping review and therefore were not performed: item 4 (online resource browsing), item 6 (contacting authors/experts), item 10 (published search filters), item 11 (adaptation from prior reviews), and item 14 (search peer review). These unexecuted items are explicitly acknowledged here to ensure full transparency in search reporting. Finally, the scoping review did not conduct a quality evaluation of the included articles, and some negative results in the study could not be clearly explained.

### Conclusions

This scoping review maps the complete research landscape for DHT-supported medication adherence targeting older adults living with chronic diseases. Distinct from previous reviews centered on effect-size pooling, this work presents an empirically induced 4-level influencing-factor framework alongside 5 age-friendly design dimensions. It extends understanding beyond simple questions of intervention efficacy toward deeper rationales concerning when interventions work, for whom they work, and how systems can be better designed. For clinical practice, nurses and interdisciplinary teams should systematically assess older adults’ digital literacy, cognitive function, and social support prior to DHT roll-out, and stratify technical-solution selection according to living-alone status. For future research, priority should be given to investigating DHT requirements among older adults who live alone or experience cognitive impairment. Empirical testing of standardized usability assessment and interoperability design is needed, alongside expanded research coverage across the full medication journey. Sustainable digital health technology for older adults does not depend on feature accumulation or fast technical iteration. Real-world success depends instead on meaningful integration with older adults’ real-life contexts, cognitive patterns, and existing social-support networks.

## Appendix

Multimedia Appendix 1 PRISMA-ScR checklist.

Multimedia Appendix 2 Search strings.

Multimedia Appendix 3 Summary characteristics of included studies.

Multimedia Appendix 4 Sunburst diagram of the 4-level influencing factor model.

## Abbreviations

DHT: digital health technology
JBI: Joanna Briggs Institute
mHealth: mobile health
PCC: population, concept, and context
P-E fit: person-environment fit
PRISMA: Preferred Reporting Items for Systematic Reviews and Meta-Analyses
PRISMA-S: Preferred Reporting Items for Systematic Reviews and Meta-Analyses Literature Search Extension
PRISMA-ScR: Preferred Reporting Items for Systematic Reviews and Meta-Analyses Extension for Scoping Reviews
SCT: social cognitive theory
UMUX: Usability Metric for User Experience

## Data Availability

All data generated or analyzed during this study are included in this published article and its supplementary information files.
