# Supplementary material for: Age-Friendly Design and Optimization of Digital Health Technologies for Medication Adherence in Older Adults With Chronic Diseases: Scoping Review
Source: J Med Internet Res. 2026 Sep 30;28:e97466. doi: 10.2196/97466 (PMC13669713; doi:10.2196/97466)
Supplement: Supplementary file 2 [file jmir_v28i1e97466_app2.docx]

**Multimedia Appendix 1- Search strings**

**Note:** This Multimedia Appendix presents the complete search strategies for all seven databases. The search was structured using the **PCC (Population, Concept, Context)** framework, as recommended by the JBI Manual for Evidence Synthesis for scoping reviews.

| **Database** | **Query** |
| --- | --- |
| Medline(via PubMed) | ((((((((((("Aged"[MeSH] OR "Geriatrics"[MeSH] OR elderly[Title/Abstract] OR geriatric[Title/Abstract] OR "older adult*"[Title/Abstract] OR "older patient*"[Title/Abstract] OR "older people"[Title/Abstract] OR "older person*"[Title/Abstract] OR senior*[Title/Abstract] OR "old age"[Title/Abstract] OR aged[Title/Abstract] OR aging[Title/Abstract] OR "older population"[Title/Abstract] OR "aging population"[Title/Abstract] OR "elderly population"[Title/Abstract]))))) AND (((((((((("Telemedicine"[MeSH] OR "Mobile Applications"[MeSH] OR "Wearable Electronic Devices"[MeSH] OR "Electronics"[MeSH] OR "Health Information Technology"[MeSH:NoExp] OR "Digital Health"[MeSH:NoExp] OR "Reminder Systems"[MeSH:NoExp] OR "Self-Help Devices"[MeSH] OR "Medical Informatics"[MeSH:NoExp] OR digital[Title/Abstract] OR ehealth[Title/Abstract] OR "e-health"[Title/Abstract] OR mhealth[Title/Abstract] OR "m-health"[Title/Abstract] OR telehealth[Title/Abstract] OR telemedicine[Title/Abstract] OR telecare[Title/Abstract] OR "mobile health"[Title/Abstract] OR "mobile app*"[Title/Abstract] OR "mobile application*"[Title/Abstract] OR smartphone[Title/Abstract] OR "smart phone"[Title/Abstract] OR "mobile phone"[Title/Abstract] OR "cell phone"[Title/Abstract] OR "electronic device*"[Title/Abstract] OR "reminder system*"[Title/Abstract] OR "electronic reminder*"[Title/Abstract] OR "smart device*"[Title/Abstract] OR "smart technology"[Title/Abstract] OR "wearable device*"[Title/Abstract] OR sensor*[Title/Abstract] OR "monitoring device*"[Title/Abstract] OR "smart pillbox*"[Title/Abstract] OR "digital intervention*"[Title/Abstract] OR "digital management"[Title/Abstract] OR "digital health"[Title/Abstract] OR "digital technology*"[Title/Abstract] OR "information technology"[Title/Abstract] OR "artificial intelligence"[Title/Abstract] OR chatbot*[Title/Abstract] OR "voice assistant*"[Title/Abstract] OR "virtual assistant*"[Title/Abstract] OR "mobile management"[Title/Abstract] OR "remote monitoring"[Title/Abstract] OR telepharmacy[Title/Abstract] OR "tele-pharmacy"[Title/Abstract] OR "remote healthcare"[Title/Abstract] OR "internet-based"[Title/Abstract] OR "web-based"[Title/Abstract] OR "smart health"[Title/Abstract] OR "connected health"[Title/Abstract] OR "digital therapeutics"[Title/Abstract] OR "clinical decision support"[Title/Abstract] OR "automated reminder*"[Title/Abstract])))))) AND (((((((((("Medication Adherence"[MeSH] OR "Medication Compliance"[MeSH] OR "Patient Compliance"[MeSH] OR "Treatment Adherence and Compliance"[MeSH] OR "Drug Therapy"[MeSH:NoExp] OR "Chronic Disease"[MeSH] OR "Chronic Condition"[MeSH:NoExp] OR medication[Title/Abstract] AND (adherence[Title/Abstract] OR compliance[Title/Abstract] OR persistence[Title/Abstract] OR concordance[Title/Abstract]) OR "medication adherence"[Title/Abstract] OR "medication compliance"[Title/Abstract] OR "drug adherence"[Title/Abstract] OR "drug compliance"[Title/Abstract] OR "treatment adherence"[Title/Abstract] OR "treatment compliance"[Title/Abstract] OR "patient compliance"[Title/Abstract] OR "adherence to medication"[Title/Abstract] OR "compliance with medication"[Title/Abstract] OR "medication taking"[Title/Abstract] OR "medication persistence"[Title/Abstract] OR "dosage adherence"[Title/Abstract] OR "dose adherence"[Title/Abstract] OR "prescription adherence"[Title/Abstract] OR "medication use"[Title/Abstract] OR "refill adherence"[Title/Abstract] OR self-management[Title/Abstract] OR "chronic disease*"[Title/Abstract] OR "chronic condition*"[Title/Abstract] OR "chronic illness*"[Title/Abstract] OR "long-term condition*"[Title/Abstract] OR comorbidity[Title/Abstract] OR "multiple chronic conditions"[Title/Abstract]))))) |
| Web of science  (Clarivate) | TS=("elderly" OR "geriatric" OR "older adult*" OR "older patient*" OR "older people" OR "older person*" OR "senior*" OR "aged" OR "aging" OR "old age" OR "older population" OR "elderly population")) AND (TS=("digital health" OR "ehealth" OR "e-health" OR "mhealth" OR "m-health" OR "mobile health" OR "mobile app*" OR "mobile application*" OR "smartphone" OR "smart phone" OR "mobile phone" OR "electronic device*" OR "reminder system*" OR "electronic reminder*" OR "smart device*" OR "smart technology" OR "wearable device*" OR "sensor*" OR "monitoring device*" OR "smart pillbox*" OR "digital intervention*" OR "digital management" OR "digital technology*" OR "information technology" OR "artificial intelligence" OR "chat-bot*" OR "voice assistant*" OR "virtual assistant*" OR "mobile management" OR "remote monitoring" OR "remote healthcare" OR "internet-based" OR "web-based" OR "smart health" OR "connected health" OR "digital therapeutics" OR "clinical decision support" OR "automated reminder*")) AND (TS=("medication adherence" OR "medication compliance" OR "drug adherence" OR "drug compliance" OR "treatment adherence" OR "treatment compliance" OR "patient adherence" OR "patient compliance" OR "adherence to medication" OR "compliance with medication" OR "medication taking" OR "medication persistence" OR "dosage adherence" OR "prescription adherence" OR "medication use" OR "self-management" OR "chronic disease*" OR "chronic condition*" OR "chronic illness*" OR "long-term condition*" OR "comorbidity" OR "multiple chronic conditions")) |
| CINAHL  (EBSCOhost) | (SU=("elderly" OR "geriatric" OR "older adults" OR "aged" OR "aging" OR "older adult" OR "older patients" OR "older people" OR "older persons" OR "seniors" OR "old age" OR "older population" OR "aging population" OR "elderly population")) AND (SU=("telemedicine" OR "mobile applications" OR "wearable electronic devices" OR "health information technology" OR "digital health" OR "ehealth" OR "e-health" OR "mhealth" OR "m-health" OR "telehealth" OR "mobile health" OR "mobile app" OR "mobile apps" OR "mobile applications" OR "smartphone" OR "smart phone" OR "mobile phone" OR "cell phone" OR "electronic devices" OR "reminder systems" OR "electronic reminders" OR "smart devices" OR "smart technology" OR "wearable devices" OR "sensors" OR "monitoring devices" OR "smart pillbox" OR "digital interventions" OR "digital management" OR "digital technologies" OR "information technology" OR "artificial intelligence" OR "chatbots" OR "voice assistants" OR "virtual assistants" OR "mobile management" OR "remote monitoring" OR "telepharmacy" OR "tele-pharmacy" OR "remote healthcare" OR "internet-based" OR "web-based" OR "smart health" OR "connected health" OR "digital therapeutics" OR "clinical decision support" OR "automated reminders")) AND (SU=("medication adherence" OR "patient compliance" OR "treatment adherence" OR "chronic disease" OR "medication compliance" OR "drug adherence" OR "drug compliance" OR "treatment compliance" OR "adherence to medication" OR "compliance with medication" OR "medication taking" OR "medication persistence" OR "prescription adherence" OR "medication use" OR "refill adherence" OR "self-management" OR "chronic diseases" OR "chronic conditions" OR "chronic illnesses" OR "long-term conditions" OR "comorbidity" OR "multiple chronic conditions")) |
| Sinomed  (China Biomedical Literature Service System) | ((TI=(elderly OR "older adults" OR "elderly patients" OR aging OR "advanced age" OR "elderly population")  AND (TI=("digital health" OR "ehealth" OR "mhealth" OR "remote monitoring" OR smartphone OR "mobile application" OR "APP" OR "smart device" OR "wearable device" OR "reminder system" OR "SMS reminder" OR WeChat OR "artificial intelligence" OR "cloud platform") AND (TI=("medication adherence" OR "treatment adherence" OR "patient compliance" OR "self-management" OR "chronic disease" OR "chronic illness" ) OR AB=("medication adherence" OR "treatment adherence" OR "patient compliance" OR "self-management" OR "chronic disease" OR "chronic illness" )) |
| China National Knowledge Infrastructure (CNKI) | SU=(elderly + "older adults" + "elderly patients" + aging + "advanced age" + "elderly population") AND SU=("digital health" + ehealth + mhealth + "remote monitoring" + smartphone + "mobile application" + APP + "smart device" + "wearable device" + "reminder system" + "SMS reminder" + WeChat + "artificial intelligence" + "cloud platform") AND (SU=("medication adherence" + "treatment adherence" + "patient compliance" + "self-management" + "chronic disease" + "chronic illness")) |
| Wanfang Data Knowledge Service Platform  (Wanfang platform) | (SU=('elderly' OR 'elderly patients' OR 'elderly population' OR 'aging population' OR 'elderly group' OR 'elderly patients with advanced age')) AND (SU=('digital health' OR 'ehealth' OR 'mobile health' OR 'telemedicine' OR 'telecare' OR 'remote monitoring' OR 'smartphone' OR 'mobile application' OR 'mobile application' OR 'APP' OR 'application' OR 'smart device' OR 'wearable device' OR 'smart pillbox' OR 'reminder system' OR 'SMS reminder' OR 'WeChat' OR 'digital management' OR 'digital technology' OR 'information technology' OR 'artificial intelligence' OR 'chatbot' OR 'Internet+' OR 'online' OR 'intelligent' OR 'cloud platform' OR 'Internet of Things')) AND (SU=('medication adherence' OR 'medication compliance' OR 'drug adherence' OR 'drug compliance' OR 'treatment adherence' OR 'patient adherence' OR 'compliance behavior' OR 'medication behavior' OR 'taking medication on time' OR 'regular medication' OR 'chronic disease' OR 'chronic condition' OR 'chronic illness' OR 'long-term disease')) |
| VIP database  (VIP platform) | (SU=('elderly' OR 'elderly patients' OR 'elderly population' OR 'advanced age' OR 'aging' OR 'aging population' OR 'oldest-old' OR 'elderly group')) AND (SU=('digital health' OR 'ehealth' OR 'mobile health' OR 'remote monitoring' OR 'smartphone' OR 'mobile application' OR 'mobile application' OR 'APP' OR 'smart device' OR 'wearable device' OR 'smart pillbox' OR 'reminder system' OR 'SMS' OR 'WeChat' OR 'digital management' OR 'information technology' OR 'artificial intelligence' OR 'Internet+' OR 'intelligent' OR 'cloud platform')) AND (SU=('medication adherence' OR 'medication compliance' OR 'drug adherence' OR 'drug compliance' OR 'treatment adherence' OR 'patient adherence' OR 'compliance behavior' OR 'chronic disease' OR 'chronic condition' OR 'chronic illness' )) |

**Taking the search conducted in PUBMED based on the PCC framework as an example:**

\# Concept 1: Population (Older adults)

"Aged"[MeSH] OR "Geriatrics"[MeSH] OR elderly[Title/Abstract] OR geriatric[Title/Abstract] OR "older adult*"[Title/Abstract] OR "older patient*"[Title/Abstract] OR "older people"[Title/Abstract] OR "older person*"[Title/Abstract] OR senior*[Title/Abstract] OR "old age"[Title/Abstract] OR aged[Title/Abstract] OR aging[Title/Abstract] OR "older population"[Title/Abstract] OR "aging population"[Title/Abstract] OR "elderly population"[Title/Abstract]

\# Concept 2: Concept (DHTs for medication adherence in chronic diseases)

"Telemedicine"[MeSH] OR "Mobile Applications"[MeSH] OR "Wearable Electronic Devices"[MeSH] OR "Electronics"[MeSH] OR "Health Information Technology"[MeSH:NoExp] OR "Digital Health"[MeSH:NoExp] OR "Reminder Systems"[MeSH:NoExp] OR "Self-Help Devices"[MeSH] OR "Medical Informatics"[MeSH:NoExp] OR digital[Title/Abstract] OR ehealth[Title/Abstract] OR "e-health"[Title/Abstract] OR mhealth[Title/Abstract] OR "m-health"[Title/Abstract] OR telehealth[Title/Abstract] OR telemedicine[Title/Abstract] OR telecare[Title/Abstract] OR "mobile health"[Title/Abstract] OR "mobile app*"[Title/Abstract] OR "mobile application*"[Title/Abstract] OR smartphone[Title/Abstract] OR "smart phone"[Title/Abstract] OR "mobile phone"[Title/Abstract] OR "cell phone"[Title/Abstract] OR "electronic device*"[Title/Abstract] OR "reminder system*"[Title/Abstract] OR "electronic reminder*"[Title/Abstract] OR "smart device*"[Title/Abstract] OR "smart technology"[Title/Abstract] OR "wearable device*"[Title/Abstract] OR sensor*[Title/Abstract] OR "monitoring device*"[Title/Abstract] OR "smart pillbox*"[Title/Abstract] OR "digital intervention*"[Title/Abstract] OR "digital management"[Title/Abstract] OR "digital health"[Title/Abstract] OR "digital technology*"[Title/Abstract] OR "information technology"[Title/Abstract] OR "artificial intelligence"[Title/Abstract] OR chatbot*[Title/Abstract] OR "voice assistant*"[Title/Abstract] OR "virtual assistant*"[Title/Abstract] OR "mobile management"[Title/Abstract] OR "remote monitoring"[Title/Abstract] OR telepharmacy[Title/Abstract] OR "tele-pharmacy"[Title/Abstract] OR "remote healthcare"[Title/Abstract] OR "internet-based"[Title/Abstract] OR "web-based"[Title/Abstract] OR "smart health"[Title/Abstract] OR "connected health"[Title/Abstract] OR "digital therapeutics"[Title/Abstract] OR "clinical decision support"[Title/Abstract] OR "automated reminder*"[Title/Abstract])))))) AND (((((((((("Medication Adherence"[MeSH] OR "Medication Compliance"[MeSH] OR "Patient Compliance"[MeSH] OR "Treatment Adherence and Compliance"[MeSH] OR "Drug Therapy"[MeSH:NoExp] OR "Chronic Disease"[MeSH] OR "Chronic Condition"[MeSH:NoExp] OR medication[Title/Abstract] AND (adherence[Title/Abstract] OR compliance[Title/Abstract] OR persistence[Title/Abstract] OR concordance[Title/Abstract]) OR "medication adherence"[Title/Abstract] OR "medication compliance"[Title/Abstract] OR "drug adherence"[Title/Abstract] OR "drug compliance"[Title/Abstract] OR "treatment adherence"[Title/Abstract] OR "treatment compliance"[Title/Abstract] OR "patient compliance"[Title/Abstract] OR "adherence to medication"[Title/Abstract] OR "compliance with medication"[Title/Abstract] OR "medication taking"[Title/Abstract] OR "medication persistence"[Title/Abstract] OR "dosage adherence"[Title/Abstract] OR "dose adherence"[Title/Abstract] OR "prescription adherence"[Title/Abstract] OR "medication use"[Title/Abstract] OR "refill adherence"[Title/Abstract] OR self-management[Title/Abstract] OR "chronic disease*"[Title/Abstract] OR "chronic condition*"[Title/Abstract] OR "chronic illness*"[Title/Abstract] OR "long-term condition*"[Title/Abstract] OR multimorbidity[Title/Abstract] OR comorbidity[Title/Abstract] OR "multiple chronic conditions"[Title/Abstract]

\# Concept 3: Context (Without any restrictions)

\# Final combination

#1 AND #2 AND #3

\# Language and publication year filters

#4 AND (english[Language] OR chinese[Language])
