# Supplementary material for: Age-Friendly Design and Optimization of Digital Health Technologies for Medication Adherence in Older Adults With Chronic Diseases: Scoping Review
Source: J Med Internet Res. 2026 Sep 30;28:e97466. doi: 10.2196/97466 (PMC13669713; doi:10.2196/97466)
Supplement: Supplementary file 3 [file jmir_v28i1e97466_app3.docx]

**Multimedia Appendix 2 Summary characteristics of included studies**

| **Author/Year** | **Aim** | **Sample** | **Study Design** | **Medication adherence assessment methods** | **Key Findings** |
| --- | --- | --- | --- | --- | --- |
| Wang M, et al.  2026 | To evaluate the effectiveness of a mobile-based video intervention ("Diabetes Little Helper") on improving medication adherence among older adults with type 2 diabetes | N=68 older adults | RCT | Self-Efficacy for Appropriate Medication Use Scale | The DLH video intervention significantly improved medication adherence among older adults, highlighting the potential of digital health tools in chronic disease management for this population |
| Wong MCS, et al.  2026 | To assess the effectiveness of the "My eDrug Manager" mobile application on medication adherence and blood pressure control among older adults with hypertension | N=569 older adults | RCT | Morisky-8 Scale | At 12 months, the intervention group had significantly higher MMAS-8 scores and a higher proportion of optimal adherence (68.6% vs. 57.6%, p = 0.007) compared to the control group, though no significant difference was found in blood pressure control |
| Bayer W, et al.  2026 | To evaluate MediTeddi, a self-determination theory-based cognitive prosthetic (social conversational electronic bear), in improving adherence and glycemic control in African American older adults with diabetes | Study 1 (N=16 older adults), Study 2 (N=50) | Mixed-method | MMAS-4 | Study 1 showed greater improvements in MMAS-4 adherence; Study 2 demonstrated that adherence significantly improved from 1.61 to 3.14, and HbA1c decreased by 1.71 percentage points, with significant reductions in depressive symptoms. |
| Mehta PK, et al.  2026 | To evaluate the impact of the AI-driven mobile application "Know Your Meds" (KYM) on drug compliance, clinical outcomes, and patient satisfaction in older adults with multimorbidity | N=360 older adults | RCT | MARS | At 12-week follow-up, medication adherence significantly improved in the intervention group compared to the control group, although no significant differences were noted in clinical outcomes (blood pressure, HbA1c, lipids) between groups |
| Amato L, et al.  2026 | To systematically assess the impact of mHealth interventions on medication adherence in older adults (≥ 65 years) with chronic diseases facing treatment burden | N=8 studies | Systematic Review | Varied across included studies | Six out of eight studies showed that mHealth interventions significantly improved adherence in the intervention group, with greater effectiveness when interventions incorporated educational and relational components beyond simple reminders |
| Wiecek E, et al.  2026 | To analyze real-world data on engagement, retention, and medication adherence among older adults (≥ 65 years) using the Perx Health mobile application | N=250 older adults | Retrospective study | Medication adherence rates were defined as the number of doses taken over the number of doses scheduled during each user's active period and averaged per day | The median medication adherence was exceptionally high at 95.0%, and older adults showed higher engagement with gamification and social features (62.5% participated in leaderboards) compared to the general user population |
| Legault J, et al.  2026 | To assess the feasibility and impact of a cognitively accessible smart clock (digital intervention) in replacing in-home medication visits for older adults with cognitive impairment in home care | N=48 older adults | Pilot study | Remote medication confirmation via the smart clock device | 72.9% of participants (n=35) successfully maintained accurate medication intake, and the intervention avoided 6,276 medication-related home visits, saving 39.6 minutes per device per day for home care staff |
| Zhang Y, et al.  2026 | To investigate the current status of medication adherence and its influencing factors in elderly tuberculosis patients managed by digital adherence technologies (DAT) | N=205 older adults | Cross-sectional study | Self-designed questionnaire survey | The adherence rate was 67.8% (139/205). Good response to digital technology follow-up (OR=0.317), high family support (OR=0.085), and mild/no dependence in daily living were protective factors, while skin allergy reactions (OR=16.357) and gastrointestinal reactions (OR=3.804) were risk factors for non-adherence |
| Wang KX, et al.  2026 | To systematically evaluate the intervention effect of mobile health management models on elderly hypertensive patients | N=30 studies | Meta-analysis | MMAS | Mobile health management significantly improved the MMAS score (SMD = 0.78, 95% CI: 0.55 to 1.02). Subgroup analysis revealed that comprehensive "Internet plus" multi-module management models were superior to single reminder-push models |
| Yu D, et al.  2025 | To evaluate the effectiveness of health management services using the "Aged Smart-Care" (ASC) mobile health platform for community-dwelling older adults | N=230 community-dwelling older adults | RCT | Morisky-8 Scale | The intervention group showed significant improvements in medication adherence, self-efficacy, and various quality of life dimensions compared to the control group |
| Yu, et al.  2025 | To Explore how the functions of mobile intervention technologies affect the management of diabetes | N=4 studies involving older adults | Meta analysis | Not mentioned | Mobile applications have been proven to improve the medication adherence and routine self-monitoring of diabetic patients. |
| Xu, et al.  2025 | Elaborate on the current situation and effect of using e-Health tools to evaluate the treatment compliance of COPD patients | N=13 studies related to medication adherence | Scoping review | Not mentioned | E-Health tools can process the medication prescription information of patients with COPD and provide personalized feedback. |
| Tuncer, et al.  2025 | To explore and summarize the effectiveness of E-health intervention measures in enhancing the medication adherence of kidney transplant patients | N=3 studies involving older adults | Meta analysis | Not mentioned | E-Health Interventions can enhance the medication adherence of kidney transplant recipients, but they need to be tailored specifically for the elderly population. |
| Sukpattanasrikul, et al.  2025 | To identify effective and cost-efficient digital health intervention measures to improve the self-management behaviors of patients with hypertension. | N=8 studies related to medication adherence | Systematic review | Not mentioned | The elderly are more active in intervention measures based on WeChat and social media. |
| Newhouse, et al.  2025 | To explore the influencing factors of e-Health tools interaction and the behavioral or attitudinal changes reported by patients themselves. | N=34 relating to the elderly and middle-aged groups | Qualitative research | Assess in the interview | Most people rely on various means such as pill boxes and mobile applications to support their medication habits, but there is still a slight lack of medication adherence. |
| Kim, et al.  2025 | To explore the demand and acceptance of digital health interventions among elderly individuals living alone, as well as the related background factors. | N=191 older adults in quantitative research and N=24 older adults in qualitative research | Mixed-method | Self-developed assessment tools | The elderly living alone are interested in e-Health information technology, but their demand and acceptance of digital health intervention measures are moderate. |
| Tang, et al.  2025 | To explore the current medication status ,influencing factors of patients with coexisting prostate cancer and its treatment. | Not application | Review | Not mentioned | Information technology can help elderly prostate cancer patients develop the habit of taking medication on time. However, the excessive pursuit of diversity and complexity in design has overlooked the actual needs and habits of the elderly population. |
| Yang, et al.  2025 | To evaluate the effectiveness and feasibility of the self-developed "Yaoshiyi App" for elderly patients with chronic diseases at home. | N=302 older adults in research | Longitudinal study | Morisky-8 Scale | Convenient and user-friendly mobile applications have the potential to enhance medication adherence among elderly patients with chronic diseases living at home. |
| Wang, et al.  2025 | To explore the application effect of cloud platforms in home care for elderly patients with coronary heart disease | N=60 older adults in research | RCT | Morisky-8 Scale | Telemedicine can enhance the medication adherence of elderly patients with coronary heart disease. |
| Yap, et al.  2024 | To analyze the effectiveness of digital health for patients with hypertension, including clinical outcomes, medication adherence, and compliance with lifestyle changes. | N=29 studies involving older adults | Meta analysis | Not mentioned | Most studies conduct patient education and medication adjustments through means such as setting up medication reminder systems, sending out educational and motivational information notifications, providing telephone education, offering remote pharmacy services, and using drugs with edible sensors. |
| Miao, et al.  2024 | To compare and rank the efficacy of various E-health intervention measures in improving the medication adherence of patients with cardiovascular diseases | N=2 studies involving older adults | Meta analysis | Not mentioned | The review found that combined interventions, telephone support, remote monitoring interventions, and application-based interventions are effective in improving medication adherence among patients with cardiovascular diseases. |
| Cao, et al.  2024 | Develop and validate a smartphone OAS app to enhance medication adherence among elderly stroke survivors | N=15 older adults in research | Pilot study | Self-developed assessment tools | OASapp offers stroke patients a more personalized and comprehensive solution through its SMS reminders, multi-user support, medication indications and visual cues, addressing their unique challenges and promoting better medication adherence among this group. |
| Badawy, et al.  2024 | To evaluate the latest evidence regarding the intensity of habits, medication adherence, and mobile health interventions based on habits in chronic diseases | N=6 studies involving older adults | Systematic review | Not mentioned | None of the included studies reported on using or proposing a habit-based mHealth behavioral intervention to promote medication adherence. |
| Al-saleh, et al.  2024 | To promote medication adherence among elderly patients with hypertension | N=26 older adults in research | Pilot study | Assess in the interview | This system may only be valuable for those elderly people who are willing to try new strategies to enhance medication adherence and utilize digital technologies. Additionally, elderly individuals have certain concerns about using smartphone applications and may require basic training in mobile device control. |
| Wang, et al.  2024 | To analyze and summarize the application of digital health technologies in medication adherence | Not application | Review | Not mentioned | Although the intelligent system has some issues, it can indeed improve improper medication behaviors and is expected to establish a new paradigm of management that shifts from passive health care to active health promotion. |
| Lu, et al.  2024 | To investigate the management efficiency of telemedicine based on the remote multiparameter monitoring in elderly patients with coronary heart disease | N=1258 older adults in research | RCT | Morisky-8 Scale | This study confirms that wearable device‑based telemedical management is an effective strategy for improving prognosis in elderly patients with coronary heart disease, particularly by enhancing medication adherence to reduce the risk of re-hospitalization. The most significant implication lies in the translation of this "digital proactive management model" from research into routine clinical practice, with the key being the establishment of an integrated remote chronic disease management system that encompasses real‑time monitoring, intelligent early warning, pharmacist intervention, and physician decision‑making. |
| Yoon, et al.  2024 | To evaluate whether smartphone app–based interventions improve medication adherence in patients with AF. | N=498  The average age of older patients with atrial fibrillation is 65.7 years old | RCT | The calculation formula is shown in the figure   | Smartphone app‑based intervention may yield tangible benefits in improving medication adherence specifically among elderly patients with higher digital literacy. However, long‑term user engagement remains to be enhanced. Future efforts should focus on precisely identifying target populations most likely to benefit, optimizing age‑friendly design of the app, and integrating multiple functions,including medication reminders, self‑monitoring, and educational feedback.So as to maximize the value of digital health tools in medication management. |
| Fu, et al.  2024 | To observe the effect of remote chronic disease pharmacy services led by clinical pharmacists on improving the medication adherence of elderly patients with hypertension. | N=198 older adults in research | RCT | Morisky-8 Scale | Remote chronic disease pharmacy services led by clinical pharmacists can enhance patients' medication adherence. |
| Zamaillo, et al.  2023 | Develop the DiabeText application, providing automated and customized short messages to support diabetes self-management. Explore the potential impact of DiabeText on self-management behaviors and blood sugar control. | N=207 older adults in quantitative research and N=89 older adults in qualitative research | Mixed-method | Assess in the interview | The SMS intervention showed no statistically significant improvement in patients' medication adherence, but the qualitative study indicated that receiving brief and frequent text messages with information related to disease management helped them remember important aspects of disease management. |
| de Souza, et al.  2023 | To analyze the effectiveness of using mobile health applications to monitor hypertension and diabetes in adult and elderly populations. | N=26 older adults in research | Systematic review | Not mentioned | Information technology has significantly enhanced patients' medication adherence. Through the use of mobile applications for the care, monitoring and management of these diseases, it helps in disease control as well as the compliance with both drug and non-drug treatments. |
| Bhagavat, et al.  2023 | To investigate the effectiveness of E-health intervention measures in improving the compliance of ACS patients with heart-protective medications | N=3 studies involving older adults | Systematic review | Not mentioned | Regardless of the type of adherence assessment method used, E-health programs do not show significantly better effects in improving the adherence to acute cardiac protection medications compared to conventional care. However, they have the potential to promote long-term secondary prevention for ACS. |
| Alves, et al.  2023 | To evaluate the impact of mobile health apps on the medication adherence of patients with heart failure | N=8 studies involving older adults | Systematic review | Not mentioned | The research results support that e-Health tools can enhance the medication adherence of patients with heart failure. However, personalized consideration is required for the elderly population. |
| YU, et al.  2023 | To explore the impact of the "Internet + nursing follow-up" model on the medication adherence and coagulation function of elderly orthopedic patients taking oral rivaroxaban | N=74 older adults in research | RCT | Judged by blood drug concentration | The "Internet + Nursing" follow-up model can regularly send messages to remind patients to take their medications, and also provide feedback to the patients, thereby enhancing their compliance and minimizing the occurrence of situations such as patients missing doses or taking the wrong medications. |
| Hui et al.  2023 | To explore the impact of the integration of internet technology and case management model on the medication adherence and blood pressure control of elderly patients with hypertension | N=190 older adults in research | RCT | Morisky-8 Scale | The integrated case management model based on internet technology can effectively enhance patients' treatment compliance and improve the long-term efficacy of medication therapy. |
| He, et al.  2023 | To explore the impact of mobile health education via mobile phones on medication adherence among elderly patients undergoing endocrine therapy for prostate cancer | N=80 older adults in research | RCT | Morisky-8 Scale | By establishing a WeChat communication group, we provided intensified education to prostate cancer patients, which significantly improved their medication adherence. |
| Van, et al.  2022 | To explore the perspectives of patients and medical practitioners regarding non-compliance with hypertension medications, as well as the potential content of digital interventions. | N=20 older adults in research | Qualitative research | Mobile applications use sensing technologies such as Wi-Fi or GPS to assess medication adherence. | For patients who do not intentionally fail to follow the treatment instructions, the intervention of information technology can effectively improve their medication adherence. However, for those who intentionally refuse to follow the treatment, the effect is rather limited. |
| Schoor, et al.  2022 | The primary aim of this study was to investigate the effect of SARA on exacerbation rates in participants with asthma and COPD. Secondary aims were to investigate its effects in terms of adherence to maintenance medication and antimycotic treatment. | N=9452 average age was 60 years old in this study. | Non-randomized Pre-Post Study | The judgment is made based on the proportion of the number of days the patient actually took the medication within a treatment cycle to the total expected medication days within the same cycle. | After SARA intervention, the medication adherence of COPD patients is usually higher than that of asthma patients, but this does not rule out that it is the result of an improvement in self-management ability. |
| Pouls, et al.  2022 | To explore the impact of using smartphones/tablets on the compliance of patients with rheumatoid arthritis in taking disease-modifying antirheumatic drugs | N=87 average age was 61 years old in this study. | RCT | The Compliance Questionnaire on Rheumatology | A serious game aimed at reinterpreting attitudes toward medication failed to show an effect on adherence to DMARDs or clinical outcomes in patients with RA. The game was played frequently indicating that it can be an effective channel for reaching patients. |
| Park  2022 | To evaluate the clinical outcomes of patients after digital intervention led by clinical pharmacists (CP) | N=8 studies involving older adults and N=6 related to medication adherence | Systematic review | Not mentioned | The impact of telephone intervention on patient outcomes varies significantly, but web-based interventions and mobile applications have generally had a positive effect. Six studies found that they improved patients' medication adherence. |
| Felker, et al.  2022 | To evaluate the effectiveness of mobile health interventions in patients with heart failure and diabetes mellitus | N=187 average age was 60 years old in this study | RCT | The Medication Adherence Self-Efficacy Scale (MASES) | In patients with HF and DM, a 3-month m-Health intervention significantly improved daily physical activity, health-related quality of life and metabolomic markers of cardiovascular health but not medication adherence |
| Emadi, et al  2022 | To determine the factors that affect the medication adherence (MA) of the elderly due to remote pharmacy services | N=52 | Scoping review | Not mentioned | Industry, policymakers, and stakeholders should consider using tele-pharmacy services for improving medication adherence among older adults |
| Yang, et al.  2022 | To explore the application effect of the Internet of Things-based health management model in elderly patients with multiple medications | N=134 older adults in research | RCT | Morisky-8 Scale | The Internet of Things-based health management model has achieved remarkable results in improving the awareness of drug-related knowledge, medication adherence, medication burden, and medication safety among elderly patients with multiple medications. |
| Wang Q  2022 | To explore the application effect of personalized blood pressure medication control plans combined with WeChat platform follow-up management on the medication adherence of elderly patients with hypertension | N=80 older adults in research | RCT | medication adherence rate = (Number of complete compliance + Number of basic compliance) / Total number of cases x 100%. | The follow-up management based on the WeChat platform is significantly superior to the conventional follow-up management. It can effectively improve the blood pressure control of patients, enhance their clinical efficacy and medication adherence. |
| Wang J  2022 | To explore the impact of information-based intervention on the medication adherence and self-management level of elderly hypertensive patients who are not hospitalized | N=186 older adults in research | RCT | Morisky-8 Scale | The information-based approach utilizes the live-streaming function of WeChat to explain the treatment and medication knowledge for elderly hypertension, thereby enhancing the understanding of the risks of the disease and the necessity of taking medication on time and in the prescribed amount. |
| Liu  2022 | To explore the impact of internet-based standardized education on the medication adherence and frailty of elderly patients with atrial fibrillation undergoing anticoagulant therapy | N=106 older adults in research | RCT | Morisky-8 Scale | Utilizing the Internet for distance education can effectively enhance the cognitive level and medication adherence of elderly patients with atrial fibrillation. |
| Chen, et al.  2022 | To explore the application effect of medication guidance based on mobile medical apps in the safe medication practice of elderly patients with chronic diseases | N=120 older adults in research | RCT | Morisky-8 Scale | The medication guidance based on mobile medical apps can improve the medication safety behaviors and medication adherence of elderly patients with chronic diseases. |
| Schulte, et al.  2021 | To explore the impact of E-health intervention on the medication adherence of patients with chronic obstructive pulmonary disease (COPD) or asthma | N=6 studies involving older adults | Systematic review | Not mentioned | The E-health intervention measures have a relatively small but significant in improving the medication adherence of asthma patients as well as those with more diverse chronic diseases and long-term medication users. |
| Maddison, et al.  2021 | To evaluate the effectiveness of text message intervention in improving the medication adherence and lifestyle changes of patients with coronary heart disease. | N=306 average age was 61 years old in this study | RCT | Morisky-8 Scale | Text4HeartII did not improve dispensed medication or adherence to a favorable lifestyle over and above usual care. This finding contrasts with previous studies and highlights that the benefits of text interventions may depend on the context in which they are used. |
| Cazeau  2021 | To summarize the research results of mobile health intervention measures in cancer patients | N=3 studies involving older adults | Review | Not mentioned | The patients were satisfied with the use of digital intervention measures and considered them helpful. However, the results of compliance varied, and multi-dimensional intervention showed better effects. |
| Capranzano, et al.  2021 | To test the feasibility of a digital task consisting in sending a QR code-based feedback of medication intake among elderly patients with heart diseases | N=262 older adults in research | Comparative study | Feedback based on QR codes | The QR code-based digital task was highly feasible for elderly with heart diseases suggesting its potential large-scale clinical application and encouraging the investigation of QR code-based systems for medication adherence telemonitoring |
| Bond, et al.  2021 | To estimate the impact of mobile health intervention on the compliance with statin drugs | N=10 studies involving older adults | Systematic review | Not mentioned | The BCTs most frequently associated with effective interventions were "Goal setting (behaviour)", "Instruction on how to perform a behaviour", and "Credible source". The combined intervention using multiple BCTs were more effective. |
| Sloots, et al.  2021 | To evaluate the patient's compliance with the E-health self-management intervention measures | N=11 average age was 71.8 years old in this study | Pilot Study | Sensorized (Respiro®) inhaler was used to analyze inhaled medication adherence | Inhaled medication adherence was high, but inhalations were poorly performed.So,personalizing and tailoring to an individual patient’s needs and competences are essential. |
| Owolabi, et al.  2020 | To assess the effect of unidirectional text messaging on adherence to dietary and activity regimens among adults living with diabetes in a rural setting of Eastern Cape, South Africa | N=215 average age was 60.64 years old in this study | RCT | Self-developed assessment tools | There is a moderate level of adherence to medication and a low level of adherence to dietary and physical activity recommendation in this setting. The text messaging intervention did not bring about any significant improvement in medication |
| Tao  2020 | To investigate the medication adherence of patients with hypertension and identify the factors that affect the medication adherence of these patients. | N=1569 more than half are elderly people and N=40 older adults in qualitative research | Mixed-research | Self-developed assessment tools | Smartphones have not been widely adopted among elderly patients, and the completion rate of follow-up was poor. This ultimately affected the effective use of mobile apps in the remote management of CHF patients, resulting in no positive outcome |
| Li, et al.  2020 | To analysis the application scheme of the joint use of WeChat platform and mobile health software in the continuous care of elderly patients with COPD | N=100 average age was 71.8 years old in this study | RCT | Morisky-8 Scale | The reminder function in the mobile app further plays a role in urging patients to engage in self-management. |
| Tu, et al.  2019 | To explore the impact of applying WeChat technology on the medication adherence and blood pressure management of middle-aged and elderly patients with hypertension | N=600 older adults in research | Comparative study | Self-developed assessment tools | Based on the WeChat platform of mobile phones, mobile medical management aimed at improving the medication adherence and management quality of patients with hypertension has become a development trend. |
| Li  2019 | To explore the feasibility of using mobile phone application platforms for remote medical management of patients with chronic heart failure | N=125 older adults in research | Comparative study | Morisky-8 Scale | Through regular follow-up via mobile app, the occurrence of situations such as patients stopping medication without permission, changing medications, or forgetting to take their drugs was significantly reduced. As a result, the medication adherence of patients with CHF was improved. |
| Su, et al.  2018 | To explore the impact of mobile APPs combined with WeChat groups on the self-efficacy, self-management, and medication adherence of elderly patients with COPD | N=96 older adults in research | Comparative study | Morisky-8 Scale | The combination of mobile phone apps and WeChat groups can enhance the medication adherence of elderly patients with COPD. |
| Yazdan, et al.  2016 | In order to better understand the issues that affect the acceptance and availability of mobile health technology (mHealth) | N=20 older adults in research | Qualitative research | Assess in the interview | The response was very positive toward the potential for a mHealth intervention to improve adherence to antihypertensives among elderly adults,but a more personalized design was required. |
| Brath, et al.  2013 | To evaluate the remote medication adherence measurement system based on mobile health (mHealth) (mAMS), which is applicable to elderly patients with increased cardiovascular risk due to diabetes, high cholesterol, and hypertension. | N=53 average age was 69.4 years old in this study | Pilot Study | Use the mAMS (Remote Medication Adherence Measurement System) | The results indicate that mHealth based adherence management is feasible and well accepted by patients with increased cardiovascular risk.e-Health blisters can be used in a multi-medication regimen but need to be carefully designed for day-to-day application |
| **Explanation of Acronyms：**  RCT：Randomized Controlled Trial；  SMS:Short Messaging Service;SARA:Service Apothecary Respiratory Advice;DMARDs:Disease Modifying Anti-Rheumatic Drugs;  RA:Rheumatoid Arthritis;HF: Heart Failure ;DM:Diabetes Mellitus;QR code:Quick Response code;BCT:Behaviour-Change Techniques；  mAMS：Remote Medication Adherence Measurement System  MMAS: Morisky Medication Adherence Scale | | | | | |
