# Supplementary material for: Age-Friendly Design and Optimization of Digital Health Technologies for Medication Adherence in Older Adults With Chronic Diseases: Scoping Review
Source: J Med Internet Res. 2026 Sep 30;28:e97466. doi: 10.2196/97466 (PMC13669713; doi:10.2196/97466)
Supplement: Supplementary file 4 [file jmir_v28i1e97466_app4.docx]

Web link for interactive sunburst diagram

<https://123456.591899.xyz/site/MultimediaAppendix.html>
